# VisTAS: blockchain-based visible and trusted remote authentication system

**DOI:** 10.7717/peerj-cs.516

**Published:** 2021-05-12

**Authors:** Ahmad Ali, Mansoor Ahmed, Abid Khan, Adeel Anjum, Muhammad Ilyas, Markus Helfert

**Affiliations:** 1 Department of Computer Science, COMSATS University Islamabad, Islamabad, Pakistan; 2 Innovation Value Institute, Maynooth University, Maynooth, Ireland; 3 Department of Computer Science, Aberystwyth University, Aberystwyth, United Kingdom; 4 Department of Computer Science and IT, University of Sargodha, Sargodha, Pakistan

**Keywords:** Deterrence, Secure authentication, Supervised authentication, Insider threats, Cryptography, Web and internet services, Data science, Databases, Security & privacy

## Abstract

The information security domain focuses on security needs at all levels in a computing environment in either the Internet of Things, Cloud Computing, Cloud of Things, or any other implementation. Data, devices, services, or applications and communication are required to be protected and provided by information security shields at all levels and in all working states. Remote authentication is required to perform different administrative operations in an information system, and Administrators have full access to the system and may pose insider threats. Superusers and administrators are the most trusted persons in an organisation. “Trust but verify” is an approach to have an eye on the superusers and administrators. Distributed ledger technology (Blockchain-based data storage) is an immutable data storage scheme and provides a built-in facility to share statistics among peers. Distributed ledgers are proposed to provide visible security and non-repudiation, which securely records administrators’ authentications requests. The presence of security, privacy, and accountability measures establish trust among its stakeholders. Securing information in an electronic data processing system is challenging, i.e., providing services and access control for the resources to only legitimate users. Authentication plays a vital role in systems’ security; therefore, authentication and identity management are the key subjects to provide information security services. The leading cause of information security breaches is the failure of identity management/authentication systems and insider threats. In this regard, visible security measures have more deterrence than other schemes. In this paper, an authentication scheme, “VisTAS,” has been introduced, which provides visible security and trusted authentication services to the tenants and keeps the records in the blockchain.

## Introduction

Authentication plays a vital role and depends on the prominence and significance of assets or resources that are being secured. Basic information systems security can be provided by implementing essential features of confidentiality, integrity, and availability (Sicari et al., 2015; Farooq et al., 2015; Kumar, Vealey & Srivastava, 2016). Implementation of managed and meticulously supervised access to its clients is the essential requirement for the security of an information system (Barrera et al., 2017). After the qualifying conditions of physical access control, next is the electronic authentication to be conducted.

Various methods are required on different IoT layers to cope with the authentication requirements in the Internet of Things (IoT). Three main layers of authentication are the system, application or program, and the users. Different architectures and policies can be implemented to accomplish IoT devices’ individuality and stop cloning the machines. The idea of using intrinsic physical characteristics of identification devices is Physical Un-clone-able Functions (PUF). This principle includes physical features at the hardware level to restrict and safeguard the device from cloning issues. Another strategy for the user or device authentication is dongle-based paired computers. Encryption schemes are incorporated in Secure authentication, which is either symmetric or asymmetric. Crossman et al. (Crossman & Liu, 2015) also recommended the use of encryption for authentication credentials as well as digital certificates issued by a Public Key Infrastructure (PKI) for secure system authentication. Similarly, the application’s authentication is accomplished using digital certificates, which is the primary solution for implementing application authentication (Markmann, Schmidt & Wählisch, 2015). After system authentication and application authentication, the authentication of users to determine their legitimacy is involved. Researchers from time-to-time have suggested different methods to minimise insider vulnerability. Most of the methods suggested the assessment and review of behavioural changes and psychological effects of the insiders. Some researchers proposed an analysis of social activities. Technical controls in this context were lacking except log analysis and tracking user activities. The Common Sense Guide and to Mitigating Insider Threats in all nations by SEI (Information Engineering Institute) provides comprehensive guidance to reduce insider threats (Silowash et al., 2012; Flynn et al., 2013). Another guide to mitigating insider risks is the Worst Practices guide by Matthew Bunn and Scott D. Sagan, who have outlined certain practices that did not prove successful (Bunn & Sagan, 2017). Various authentication schemes are used for the authenticity of a user. The following section will discuss threats & vulnerabilities, authentication procedures, and various authentication schemes.

Temporal variables are additional parameters for authentication security. (i) Something you know (ii) something you have and (iii) something you are, are some known identification variables. Similarly, temporal variables to constraint an individual are (i) only for Identified User, (ii) only for Specified Time, and (iii) only at specified Geo-Location (Ali et al., 2020).

**Figure 1:**
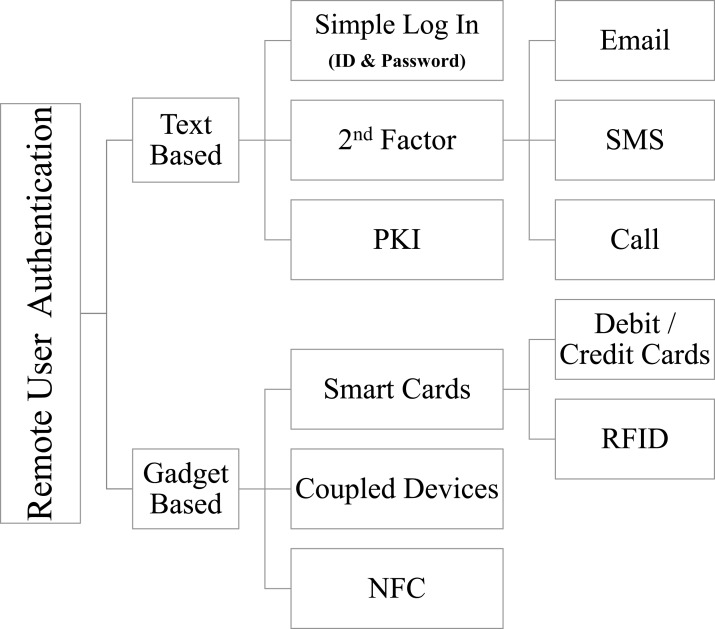
Remote user authentication—taxonomy.

Multiple types and methods are used to provide user authentication, e.g., one factor uses user ID and password. The 2^*nd*^ factor is used for additional security, such as verification of authentication via SMS, use of biometric devices, passcode via email, or even using a phone call or any other appropriate multi-factor authentication schemes. The 3^*rd*^ element for secure authentication is the use of encryption techniques in the transfer of credentials. Credit/Debit Card Transactions are validated by a three-dimensional (3D) authentication, which depends on another party for a secure authentication approach. In the delivery of secure authentication, smart cards often play a significant role. Users can be identified and authenticated remotely by various methods as shown in Fig. 1. A detailed taxonomy as developed in Ali et al. (2020) covers various other authentication schemes.

- Text-based authentication, which uses textual input (user identification and password), which may include Multi-factor Authentication using SMS, email, etc., also).
- 3rd dimensional (the other party guaranty is essential for transactions on financial cards).
- Biometrics (human body sensors like fingerprints, voice signatures, retina scans, and other wearable sensors).
- Coupled dongles (devices like electronic gadgets etc.).

We need to concentrate not just on identification by recognition but also on verification and authentication of each operation and transaction in terms of financial services and other sensitive environments. IoT operating system based centralised authentication is another concept to allow access control. The growth, availability, and application of these newly introduced operating systems would dramatically improve IoT users’ confidence. The WoT operating system would also increase users’ trust in WoT, i.e., IoT scalability, to a higher degree. Users belong to various set-ups and are granted varying types of access permissions (Ahmad et al., 2016).

### Distributed ledger technology’s advantages

A blockchain is a distributed ledger composed of blocks that are linked with the help of cryptography. Each block is made up of a sequence of transactions. To secure the entire chain of these blocks, each one is linked to its predecessor using a cryptographic hash. Even in a decentralised environment, data stored in a blockchain can be verified, leading to a wide range of blockchain applications. Chowdhury et al. (2019) and Deepa et al. (2020) have presented a comparative analysis that reviews the viability, approaches and opportunities of the well-established DLT platforms, both private and public. Anyone can change the state of a public ledger (Permissionless Ledger) by storing new blocks and updating data through transactions between participating entities. In contrast, only authorised and trusted entities can participate in transactions on a private ledger (Permissioned Ledger), ensuring that the ledger’s data is kept private. Smart contracts are used to keep transaction processes secure and traceable (Bhardwaj et al., 2020). Multiple mission-critical applications have implemented blockchain-based secure data delivery and storage as discussed by Bera et al. (2020) and Ali et al. (2021). The benefits and mechanism of data storage in the blockchain is discussed by Wang et al. (2020). Along with the advantages of blockchains, this technology is not yet mature enough to handle security, privacy and management issues completely, as highlighted by Singh et al. (2020) and Singh, Hosen & Yoon (2021).

### Motivation

Trust measurement and incident monitoring are used to perform trust evaluations. Computing environments are very dynamic and versatile in nature. Hardware, Software, Databases, and Communication threats collectively pose to IoT environment as highlighted by Bou-Harb et al. (2017); Fischer (2014) and Langner, 2011; Masdari & Ahmadzadeh (2017). Multidimensional threats have been reported that pose to Security and Privacy separately and Trust in IoT as a whole. A taxonomy of basic security threats is explained by Jouini, Rabai & Aissa (2014). Most Information Technology infrastructure components are vulnerable to a wide range of threats (Bisong & Rahman, 2011; Kandias, Virvilis & Gritzalis, 2011; Schlicher, MacIntyre & Abercrombie, 2016). These can be categorised into external or outsider threats as well as internal threats or insider threats.

- **Outsiders Threat:** Also known as External Threat. A threat originating from outside of a company, government agency, or institution Jouini, Rabai & Aissa (2014).
- **Insiders Threat:** This is also known as the inner threat or internal threat. Threat originating within a company, government agency, or institution and typically exploited by a disgruntled employee denied promotion or informed of the termination of employment (Jouini, Rabai & Aissa, 2014). Insiders could have direct access to the organization’s ICT infrastructure and can exploit the vulnerabilities, and may have escalated privileges by breaching the information protection control system (Yusop & Abawajy (2014).

Ivan Homoliak has conducted a state-of-the-art survey (Homoliak et al., 2019) and compiled all the available dimensions of insider threats and defence solutions in this category. Though different types and factors-based authentication schemes have been proposed, as summarised in Table 1, all of them have limitations to provide supervised authentication, peer control, visible access, storage of immutable login information, and deterrence.

**Table 1:** Authentication types.

Sr. No	Factor(s)	Initial Parameters	Mutual Authentication	Applicability	Attacks Covered	Vulnerabilities	Threats	Model	Vulnerabilities
1	1FA	User ID & Password	No	Very easy and user friendly	Open Access, Basic Identity	Multiple	Multiple	Simple Login Form	One Time ID/Password may be guessed/cracked
2		Use of Biometric Authentication	No	Difficult, Biometric devices are not available anywhere	Social engineering/dictionary	NO	NO	Finger Print Readers	Online Systems are not matured enough
3		Use of wearable sensors (ECG, EEG)	No	Difficult, Devices are not available anywhere	Social engineering/dictionary	NO	NO	Medical Gadgets	Online Systems are not matured enough
4		Voice signatures	Yes	Difficult, Devices are not available anywhere	Social engineering/dictionary	Voice Signature Reproduction	Yes	Google Voice, Nuance etc.	Recorded data can be reproduced easily
5	2FA	User ID & Password, Email is used for 2FA	Yes	Very easy and user friendly	User Identification & Mutual Authentication	Email Spams	Email may already Compromised	Financial Transactions	If email accountis already compromised
6		User ID & Password, Mobile Phone is used for 2FA	Yes	Very easy and user friendly	User Identification & Mutual Authentication	Smart Phone Vulnerabilities	Phone may already Compromised	Email Services like Gmail	If Mobile Phone is already compromised
7		User ID & Password, USB is used for 2FA	Yes	Very easy and user friendly	User Identification & Mutual Authentication	Smart Phone Vulnerabilities	Phone may already Compromised	Gmail USB Dongle based Authentication Services	What if USB Dongle Got Lost/Cloned
8	3FA	User ID & Password, Email is used for 2FA, Symmetric encryption to avoid spams is incorporated	Yes	Easy, but technically depends on user skill level	User Identification & Mutual Authentication	Key Compromises	–	Custom Build Authentication Frameworks. e.g., WebSeA	If any of the factor source/services are not available
9		User ID & Password, Email is used for 2FA, Asymmetric encryption is incorporated	Yes	Technically depends on user skill level	Non-repudiation	Certificate may Lost	–	Custom Build Authentication Frameworks. e.g., WebSeA	If any of the factor source/services are not available
10	3D	3rd party acts as intermediator	Yes	Requires trust among parties	Non-repudiation	International laws may not be effective		International joint Ventures like VISA and Master	A Central DRU (Dispute Resolution Unit) Acts to resolve the issues
Additional Authentication Factors (AF)									
11	4th AF	Geo Location parameter	Yes	Not applicable for indoor activities	–	Services may not be available everywhere	IP Based Location Tracking	EBSCO Services (SANS Patent)	IP Cloning/Spoofing etc.
12	5th AF	Voice signatures	No	Easy, but technically depends on user skill level	–	Recorded data can be reproduced easily	Sound may vary due to weather and may be reproduced	Google Voice, Nuance etc	

### Contribution

Insider threat is a fundamental and important cause of data breaches. A Russian proverb has fascinating stories about the USA—Russia relationships as “Trust, but Verify” Markóczy (2003). Some psychologists counter it as “Distrust and vilify”, a totally different approach. A sharp liner difference exists between vigilance and distrust, i.e., both are very different.

Whereas, to develop mechanisms that can protect systems from such insider threats, we have to reconsider the importance of vigilance (Markóczy, 2003). Institutes engage employees because they have trust in them. Identification and authentication mechanisms focus on user input, but ID theft and password leakage, social engineering are common practices in people with malicious intentions. In this paper, we have proposed a visible monitoring system to mitigate insider threats. Physical, logical, and social levels should be considered to analyse the insider threat holistically to prevent, detect and recover from these attacks. Our primary focus is on how to allow privileged users to perform valid/legitimate activities only. Other layers of information security should also be considered carefully. The contribution and research questions are as under.

**Research Questions:** A generic information security system is supposed to provide deterrence against miscreants’ attempts, prevent and protect from their attacks, timely detect such attempts and finally provide remedies against such detected abusive acts. Validation of any transaction in Information System Management operations/activities is critical and will enhance the system security exponentially (Theoharidou et al., 2005). Identity management and authentication schemes are the core area of a secure information system. Though different types and factors based authentication schemes are present, as discussed earlier but all of them failed to provide supervised authentication, peer control, visible access, and deterrence (Homoliak et al., 2019). Research questions have been formulated after a comprehensive literature survey, and “Deterrence” is found as the only research area left behind which needs more focus.

1. How to achieve deterrence in information security (Fear of being caught red-handed)?
2. How to provide visibility in an authentication scheme?
3. How to achieve peers confidence for better trust?

This paper has proposed a deterrence-based authentication system in which authentication is carried out in a peer review and visible to the stale holders. The login requests are recorded in a distributed ledger and shared among registered peers.

Related work concerning authentication schemes is covered in “Related Work”. Highlights of contribution of this paper is given in “Contribution” and proposed authentication system (VisTAS) is covered in “The Proposed Model—VisTAS”. The performance and results of the proposed system are explained in “Results & Discussion”. SWOT analysis containing Strengths, weaknesses, opportunities, threats, a summary of results and discussion of the proposed model have been covered in “Swot Analysis”, and the paper is finally concluded in “Conclusion and Future Work”.

## Related Work

Electronic authentication evolution is revolutionary and has reached the current state through several improvements to provide security to the resources. With the advent of technology and hacktivism, the initial text-based single-factor authentication scheme could not meet the information security requirements. This led to the development of two-factor authentication and further progress in using multi-factor encryption and coupled devices. Wang et al. Wang et al. (2015) published a systematic study of two-factor authentication in which authors have posed concerns and weaknesses in the two-factor authentication mechanism and showed uncontrollable issues with the functional manifestation of adversary resources. Since the introduction of two-factor authentication, the usage of coupled sensors has been implemented. Van der Haar et al. studied the critical implementation of the recursive utility of smart devices. IoT requires authentication for the protection, services, and advantages of utilising wearable sensors to authenticate individuals protected by this article. IoT is recursively applied, e.g., IoT also requires authorisation as these are required for the authentication of wearable sensors (van der Haar, 2015). Munch-Ellingsen et al. boosted their opinion of 2^*nd*^ factor authentication by utilising coupled/hardware-based authentication. Cipurse contactless cards were initially developed to satisfy the transport industry’s needs, and the first iterations of the specification were mirrored and followed by the Open Standard for Public Transport (OSPT) Alliance. Since smartphones are mainly Bluetooth-enabled, Bluetooth-based devices are proposed to monitor smartphones as coupled devices (Jeong et al., 2015). Host Card Emulation (HCE) and Close Field Contact (NFC) are the two aspects of smartphone-based authentication. They have a single element of IoT authentication. The writers have illustrated the limitations and abuse of these functions.

An additional SMS service solution was proposed as a 2^*nd*^ factor (Munch-Ellingsen et al., 2015) authentication to fix these flaws. The user’s security and data privacy risks are outlined in Jacobsson’s home automation systems and explored in the Smart Homes (Jacobsson, Boldt & Carlsson, 2015) realm. All the research culminated on the importance of integrating security and privacy into the design phase of any new development (Jacobsson, Boldt & Carlsson, 2015). Impersonation, repeat, and related attacks are typical to OAuth. Work has been done to resolve these issues by incorporating another principle of Security Manager (Emerson et al., 2015). This Security Manager enhances security, availability, and efficiency by utilising a database recording the expiry time tokens, including other useful information to reduce various IoT network registrations and numerous IoT network logins.

Arno et al. developed the idea of engaging smart devices for securing assets and proposed a smart lock for bicycles using smartphones (Arno, Toyoda & Sasase, 2015). The central idea of this lock is an accelerometer-based authentication. Sample data is produced using the NFC, GPS, Bluetooth devices, and the idea is executed by using an Android-based smartphone (Munch-Ellingsen et al., 2015). User authentication is required when Cloud and IoT service providers need periodic access to IoT/Smart Devices for firmware upgrades and other routine maintenance (Barreto et al., 2015). The idea of using Dynamic ID is quite active now, and a study is underway to secure IoT using Dynamic IDs (Zhai et al., 2015; Ilyas, Ali & Kueng, 2010). The IoT mutual authentication system based on login ID, password hash, and MAC address along with the DBMS (Database Management System) for the management and logging of authorised and unauthorised access controls is proposed (Devi et al., 2015). Ruan et al. raised concerns about misuse of identity. Impersonation attacks are very popular in identity misuse. A random oracle framework is designed to counter the impersonation attack by widening the two-party conuration to the ’n’ parties and developing an efficient two-party EAKA (explicit authentication key agreement) protocol as provided by the standard (Ruan et al., 2015) model.

Delegation-based IoT authentication is proposed in Borgohain et al. (2015) to resolve privacy concerns in IoT. The private mutual authentication model is introduced, which uses PKI encryption schemes to respond to privacy and security concerns using new protocols. The research introduced Identity-based Cryptography (IBC) and Elliptical Curve Cryptography (ECC) for end-to-end authentication. Asymmetric cryptography for end-to-end encryption (Markmann, Schmidt & Wählisch, 2015). Integrating Ciphering and Physical Authentication schemes is suggested for additional security, and 3rd-factor authentication Delegation-based IoT authentication is proposed in (Borgohain et al., 2015) to resolve privacy concerns in IoT. Authors have implemented this system using the open-source Vanadium framework (Wu et al., 2016). The research introduced Identity-based Cryptography (IBC) and Elliptical Curve Cryptography (ECC) for end-to-end authentication. Asymmetric cryptography for end-to-end encryption (Markmann, Schmidt & Wählisch, 2015). Integrating Ciphering and Physical Authentication schemes is suggested for further security and 3rd-factor authentication (Crossman & Liu, 2015). It is also highlighted that desirable security objectives can be obtained by providing Dynamic IDs-based authentication. The advanced framework for communicating multi-site knowledge with Ciphered Dynamic credential is also demonstrated in Ilyas, Ali & Kueng (2010). The IoT Continuous Authentication Protocol, where smart devices frequently communicate limited data/messages at short intervals, is proposed by Bamasag et al. The protocol is based on the Shamir secret sharing system, with the innovation of mutual authentication. Claimer Identity is checked using tokens provided for the same function (Bamasag & Youcef-Toumi, 2015; Crossman & Liu, 2015). It is also highlighted that desirable security objectives can be obtained by providing Dynamic IDs-based authentication. The advanced framework for communicating multi-site knowledge with Ciphered Dynamic credential is also demonstrated in Ilyas, Ali & Kueng (2010). The IoT Continuous Authentication Protocol, where smart devices frequently communicate limited data/messages at short intervals, is proposed by Bamasag et al. The protocol is based on the Shamir secret sharing system, with the innovation of mutual authentication. Claimer Identity is checked using tokens provided for the same function (Bamasag & Youcef-Toumi, 2015). Developing modern protocols and integrating new features of IPV6 and 5G connectivity into IoT has been proposed by Mahmoud et al. (2015). The new word “Threat Index” is introduced to measure vulnerabilities in IoT and recommends the creation of new protection approaches for each layer of IoT (Kumar, Vealey & Srivastava, 2016). In the usage of IoT, the privacy of a person remains at risk. With the introduction of smart technology, it is really important to take account of consumer safety. Often RFIDs are used for recognition purposes in IoT. The authors recommended the usage of IPSec along with RFID to protect user privacy. In this method, “Need to Know” dependent rule is implemented (Gross et al., 2015). Quick reply with IoT devices certainly improves every machine’s performance, but it again requires so much caution as suggested in (Condry & Nelson, 2016). A lightweight anonymous authentication protocol is recommended for an RFID-dependent authentication. In this technique, random tokens are created to preserve user privacy. It is claimed to protect consumer privacy, whereas cryptographic functions are neglected, which will face certain serious threats of misuse of RFIDs (Chen, Chen & Fang, 2017). Similarly, Díaz et al. implemented the Zero Information Authentication Protocol concept, along with several other authentication factors in IoT authentication. One time password (OTP) and Short Message Service (SMS) are two other variables that can be used for authentication (Díaz, Martn & Rubio, 2016), and (Jun, 2010). The session period plays a critical role in the system’s security. Authentication for a restricted period would exponentially impact systems’ security (Barrera et al., 2017). An important way is to handle authentication with restricted/limited information sharing or nil knowledge sharing as described in Coffey & Newe (1998). A new solution of hybrid cards (Swing-Pay) is presented, where a digital card unit comprising NFC and bio-metric authentication for peer-to-peer payments and identity management (Ghosh et al., 2017). Different forms of authentications are introduced to improve the process. Similarly, another Protocol (Pay-Cloak) (Majumder et al., 2017) to perform internet purchases using a bio-metric back cover for mobile phones has been suggested. Signature dependent authentication is indicated in Nishigori, Kawamoto & Sakurai (2017) where biometric grid reference points of an individual’s signature and the other behavioural characteristics of the human-being are analysed for secure authentication. These behavioural characteristics include writing pace, pen pressure on the paper, angle of the pen. Shoulder surfing attack and the availability of a printed copy of the user’s signature can dodge the scheme. Secure Authentication in Industrial IoT as proposed by Xiaoding et al. (2021) manages a user’s access to the blockchain as well as other applications. Another, very recent three-factor remote user authentication has been proposed by Patel et al. (2020) in which a record of authentication requests is controlled by an administrator or superuser. Rathee et al. (2020) proposed a graph-based social network model for forensic perspectives. Jiang et al. (2019) proposed an authentication protocol that uses an identity-based cryptosystem in which public key is used as the user’s identity, eliminates the need for certificates and simplifies network configuration, which is very useful for a common user instead of an administrator or superuser. Authentication and insider threat are crucial issues, and research is in progress where different methods are proposed and practised for securing an information system.

## The Proposed Model—VisTAS

A closed and confined environment is proposed where a digital fence in terms of IDS (Intrusion Detection System), and the IPS (Intrusion Prevention System) systems, are implemented to provide the information security of a mission-critical system. Much research has been carried out to implement cybersecurity measures for physical threats from insiders, such as limited access, constant monitoring with close circuit cameras, and 24/7 surveillance by overlapping physical security personnel. Data transport is another critical issue where a man in the middle (MITM) attack can be used to steal data from insiders, and therefore protection must be carried out in this regard. Smart cryptography techniques for the secure distribution of larger data in cloud computing should be applied.

### Access types

Different level access permissions are required to different levels and categories of the users. These can be categorised as follow:

**Administrative Access:-** Network operations, system administration & maintenance, backup & restore operations, database administration, and Data Transportation are such activities that are considered administrative activities and need escalated privileges.

**User level Access or limited access:-** Software usage, simple desk-work, printing, internet surfing are known as user activities that do not require escalated privileges than a standard user.

### Experimental evaluation and environment setup

The proposed framework uses multi-factor authentication in a supervisory concept and multichain based blockchain for the immutable storage of access requests to mitigate the insider threat. For a Proof of Concept (POC), We have deployed VisTAS on a CentOS Server. The detail of the resources is as under.

**Hardware Resources** configured for the implementation and testing of the proposed VisTAS include a Dual Core CPU with 100 % Execution Capacity, 4 Gigabytes for Random Access Memory, 20 Gigabytes HDD for installation & storage, and a bridged network adopter.

**Software Resources** used to implement and test VisTAS in Linux using CentOS 8.0 x86_64 for the above-discussed hardware resources. Multichain 2.0.0 is used for blockchain implementation based on The Elliptic Curve Digital Signature Algorithm (ECDSA). We used Elasticsearch with Kibana in a Firefox 73.0.1 browser interface for storage and query of the data.

### Implementation

Close circuit television cameras (CCTVs) are installed for physical monitoring and local login requests, and screen activities are also recorded using screen capturing applications. These activities are discussed in the following sections.

### Operational sequence

**Figure 2:**
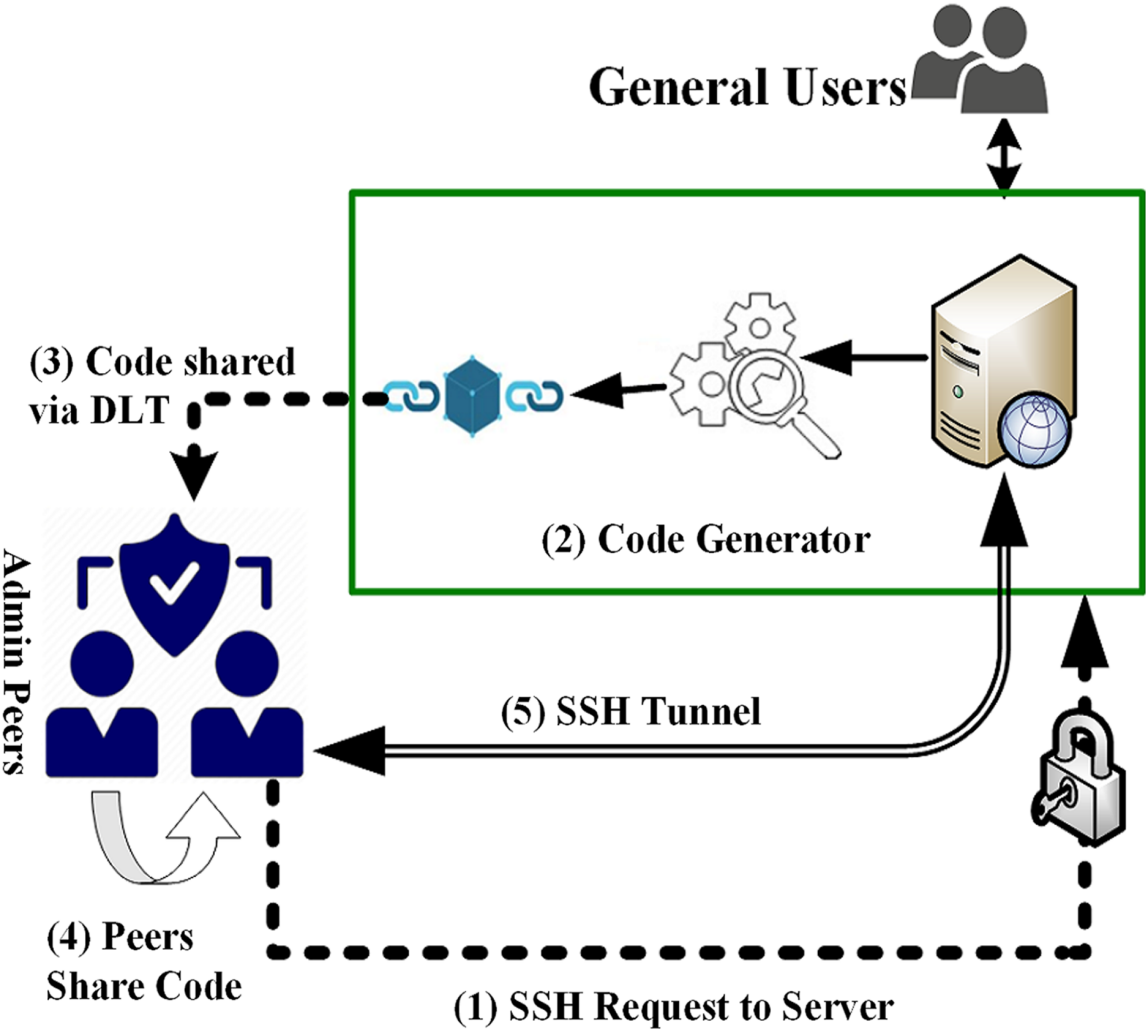
VisTAS—proposed authentication architecture.

This framework is covered in two different types of activities, i.e., Admin Activities and User Activities. Workflow and sequence of the activities are given in Fig. 2 and enlisted as under.

- Administrator attempts to get the login to the server remotely using SSH (secure shell in Linux servers) and enters his/her ID and password.
- Server generates a random token, records it along with the request into a distributed ledger (DLT), and sends it to the supervisor/peer/colleague (as desired by the organization).
- Upon verification of the credentials, Requesting administrator is granted access to predefined resources/services.
- All the authentication credentials are recorded in a DLT and vet by the other peers.
- These entries are visible to peers and are being recorded in a DLT.

**Figure 3:**
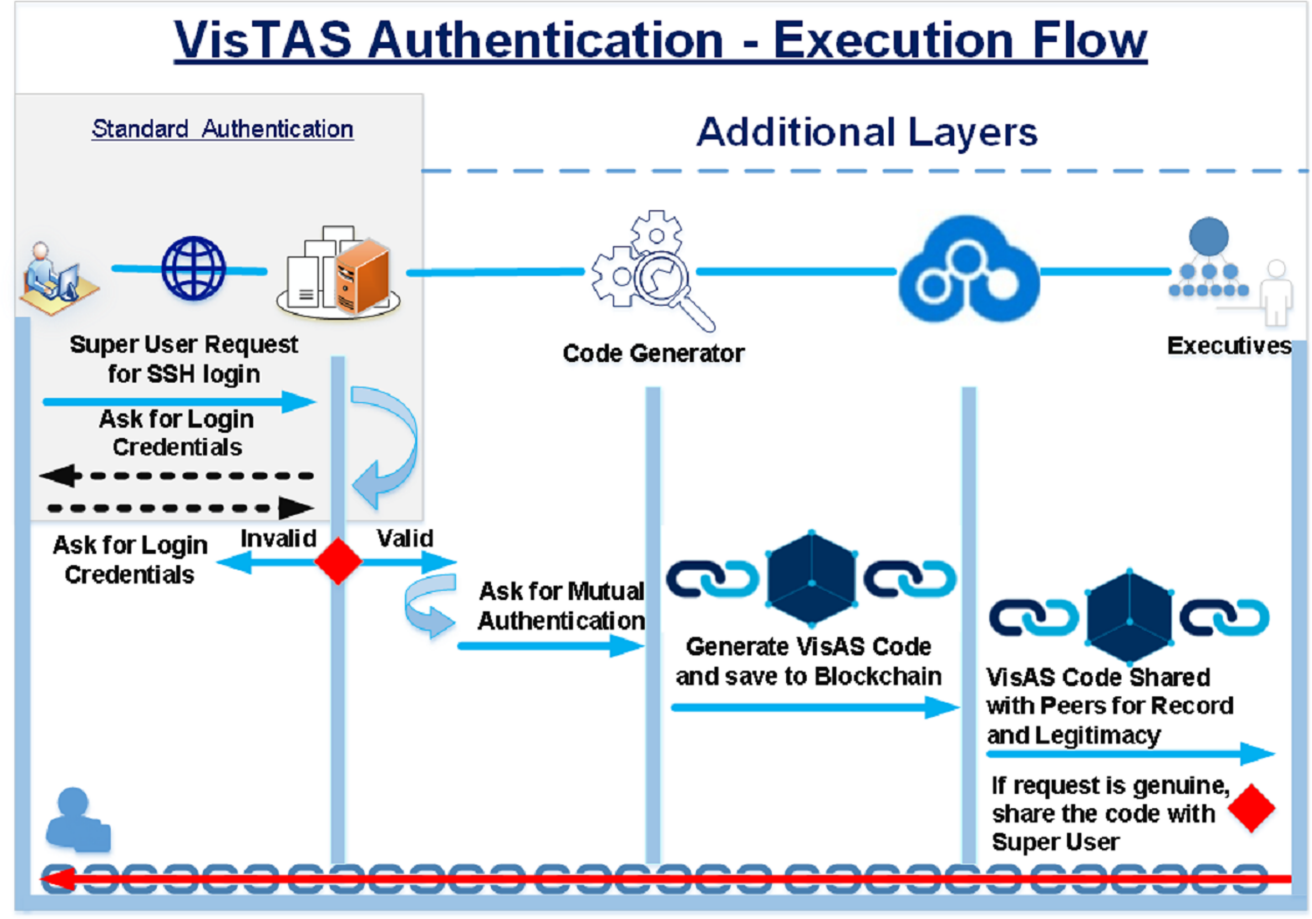
Execution flow of proposed architecture.

The contrast of standard authentication and additional authentication carried out by VisTAS is shown in Fig. 3 and Table 2 where a random code generator and blockchain has been introduced for visibility and transparency.

**Table 2:** A contrast of authentication schemes.

Ser.	Parameter	Existing schemes	Proposed scheme
1	Confidentiality	Simple data transfer	Encrypted data transfer
2	Availability	Existing authentication schemes does not confirm availability	Our system provide higher fault tolerance in terms of availability by denying illegal requests
3	Immutability	Existing authentication schemes do not provide integration with blockchains	Proposed authentication scheme provides integration with blockchains
4	Traceability	Existing authentication schemes have no traceability mechanism using immutable data structures i.e., blockchains	Proposed scheme store traceable data in blockchaines to provide immutability
5	Speed	Data transmission occure at the network speed	A very negligible transmission delay may occure while storing data in the blockchain
6	Transparency/visibility	Peers and other members have to visibility on the authentication system	Peers have access on the blockchain streams to view data and allow and validate authentication
7	Mutual Authentication	No practice of mutual authentication	Mutual authentication is carried out using two men rule

## Results and discussion

Performance and security are two very important factors in a server’s health. However, security becomes a critical objective that can supersede any performance matrix, and that’s why sometimes we have to compromise on performance to achieve security. The effectiveness and workload of any framework or scheme can be obtained by monitoring the server on multiple parameters, especially the usage of processing power, IO activities, random access memory, network utilization, swap memory, and context switching. It is a win-win situation if the system/facility becomes deterrent/secure without compromising these performance matrices significantly. There are multiple application and system monitoring tools freely available, whereas System Activity Reports. Comparisons of these performance matrices are shown in the following graphs. SAR is one of the utilities being provided by “sysstat” which is a linux package bundled with other different utilities for system performance review. SAR utility can provide the following types of statistics to monitor and evaluate a system state.

- The overall CPU usage or workload
- Individualist CPU statistics
- Memory status (how much used and remaining available)
- Swap space status (used and available)
- I/O activities (System Wholesome)
- I/O activities (Individual Devices)
- Operating system statistics for context switching
- Load average and running queue data
- Statistics providing network status
- Specific interval report of SAR data

In this research, we used this utility to generate performance comparison on standard authentication viz-a-viz VisTAS based authentication. We focused on monitoring only important performance matrices like CPU Usage, Memory Usage, IO load, swap memory usage, context switching, process queue, and network traffic only.

### Complexity and efficiency

The most important segment of VisTAS is storing data in a blockchain which is carried through APIs. As discussed in the previous section, VisTAS used “Multichain”, and these APIs have a time complexity of O(log(n)), where n is the number of items being stored or retrieved (Multichain, 2019). APIs use index lookups in retrieving a data block and any general index has a complexity of O(log(n)). The efficiency and processing impact of the proposed model is covered in subsequent sections.

### I/O load analysis

Input-Output (I/O) workload has a significant impact on the performance of a computing system. The following parameters can be used to evaluate I/O overhead using SAR in the context of I/O and transfer rate statistics. VisTAS requires additional overhead for data input and processing for additional security.

- **tps** The total number of transfers issued per second to physical devices. A transfer is a request for an I/O to a physical device. Multiple logical requests can be merged and treated as a single device I/O request. The data transfer can be of an undetermined volume.
- **rtps** The total number of reading requests to physical devices issued per second.
- **wtps** The total number of write requests issued to physical devices per second.
- **bread/s** The total volume of data read received the devices in blocks per second. Blocks are equivalent to sectors with 2.4 and newer kernels; therefore have a size of 512 bytes. With older kernels, the block is of an undetermined size.
- **bwrtn/s** The total amount of data written to devices in blocks per second.

**Figure 4:**
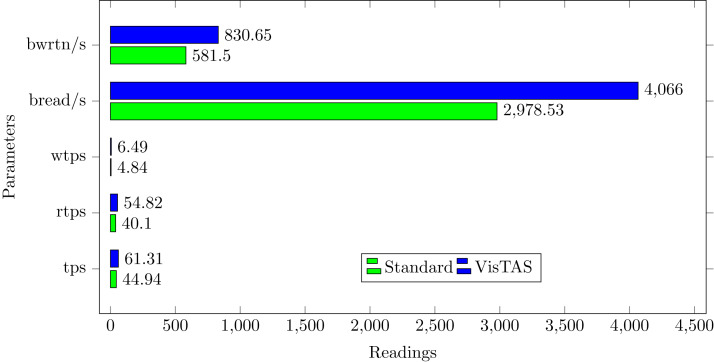
I/O load comparison.

I/O overhead shown by the SAR statistics using the VisTAS as compared to the standard is shown in Fig. 4.

### CPU usage analysis

CPU usage analysis return the statistics for the following:

- **%usr**: The utilisation of the CPU in terms of percentage occurred during the execution of a user-level application.
- **%nice** The utilisation in percentage that occurred during the execution of a user-level application with nice priority.
- **%system** The CPU utilisation percentage that occurred in the execution of a system level (kernel) activity which also includes the time spent in servicing the hardware and software interrupts.
- **%sys**The CPU utilisation in percentage that occurred while running at the system level (kernel) excluding time spent on the hardware or software-based interrupts.
- **%iowait** The percentage of the time that a CPU or CPUs were idle during which the system had an exceptional I/O disk request.

**Figure 5:**
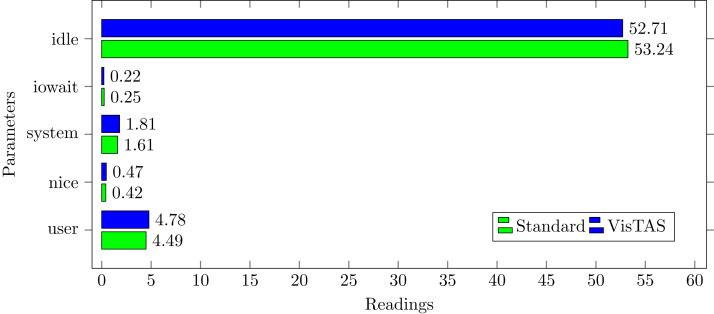
CPU usage.

By analysing the graph shown in Fig. 5, processing impact is very minute while using VisTAS compared to standard authentication.

### Context switching analysis

**Figure 6:**
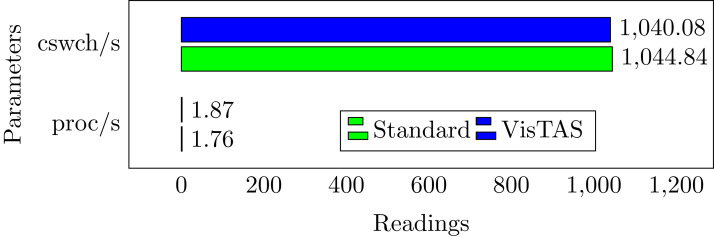
Context switching.

Context Switching leads to an additional workload associated with sharing the device cache for various tasks, running the scheduler. Context switching among the threads of the same application or process is faster than different processes. Its overhead can be observed by the process created per unit of time and the number of context switches that occurred per second as follows, and the comparison is shown in Fig. 6.

- **proc/s.** The total number of tasks created in a unit time.
- **cswch/s.** The total number context switches occurred in a unit time.

### Process queue analysis

Statistics showing the length of the system process’s queue and load averages determine the system’s efficiency. The following can be used to check the system’s health and performance:

- **runq-sz.** This shows the number of tasks waiting for the CPU.
- **plist-sz.** Returns the total number of the tasks present in the task list.
- **ldavg-1.** Returns the last-minute average load.
- **ldavg-5.** Returns the average system load for 5 min.
- **ldavg-15.** Returns the average system load for last 15 min.

**Figure 7:**
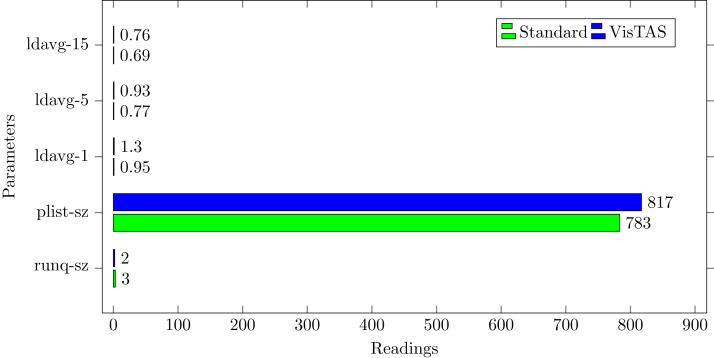
Queue impact.

The impact of VisTAS on process queue management compared to standard authentication as depicted in Fig. 7 is negligible.

### Network traffic analysis

- **rxpck/s.** Shows total number of packets received in unit time.
- **txpck/s.** Shows total number of packets transmitted in unit time.
- **rxkB/s**. Shows data volume in kilobytes received in unit time.
- **txkB/s.** Shows data volume in kilobytes transmitted in unit time.
- **rxcmp/s.** Shows compressed packets count received per second (for cslip etc.).
- **txcmp/s.** Shows compressed packets count transmitted per second.
- **rxmcst/s.** Shows multi-cast packets count received per second.

**Figure 8:**
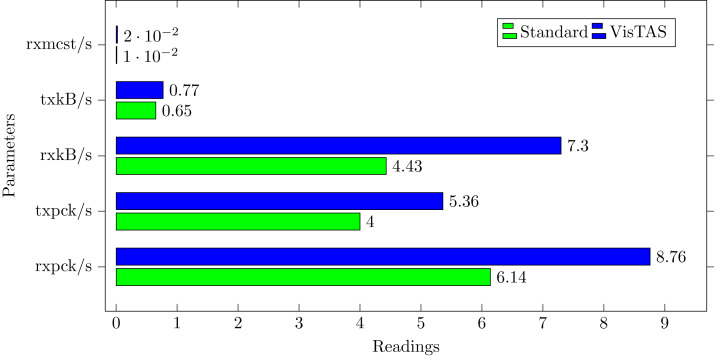
Network load analysis.

While comparing network load between VisTAS and standard authentication as shown in Fig. 8, it observed a difference of two to three packets for an authentication activity.

### Swap memory usage analysis

Swap memory extends system memory. The utilisation of this memory is also used to monitor the system’s performance. Important attributes to monitor swap memory are as follows.

- **kbswpfree.** Free swap space available in kilobytes.
- **kbswpused.** Used/Occupied swap space in kilobytes.
- **%swpused.** Percentage of used swap space.
- **kbswpcad.** Cached swap memory in kilobytes. This is the memory swapped out earlier and is swapped back in but still also in the swap area.
- **%swpcad.** Percentage of cached swap memory in relation to the amount of used swap space.

**Figure 9:**
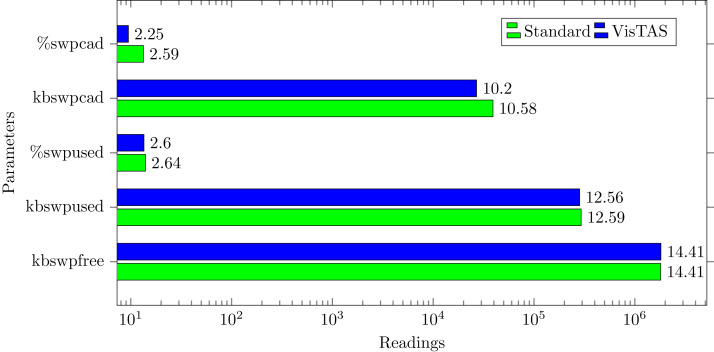
Swap memory load.

Figure 9 has shown the swap memory impact comparison between VisTAS and standard authentication and ignore-able difference found in swap memory utilization.

### Memory usage analysis

**Figure 10:**
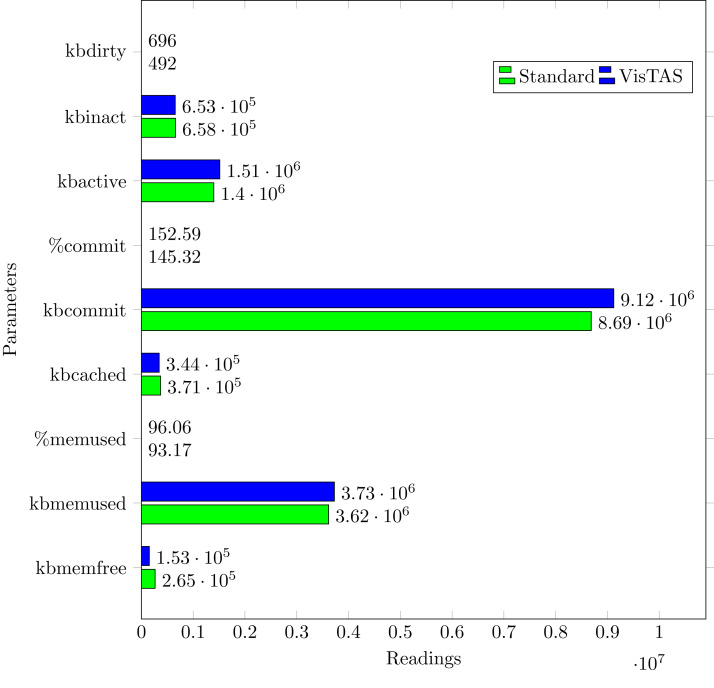
Overall memory usage.

Memory usage analysis is carried out as shown in Fig. 10 in terms of the following:

- **kbmemfree.** Available free memory (kilobytes).
- **kbmemused.** memory used (kilobytes). Excluding the memory kernel itself used.
- **%memused.** Used memory in percentage.
- **kbbuffers.** Buffer memory used by the kernel in kilobytes.
- **kbcached.** Cache memory used by the kernel in kilobytes.
- **kbcommit.** Memory needed for the current workload (kilobytes). An estimate of how much RAM/swap is required to guarantee that system never goes out of memory.
- **%commit** Memory percentage required for the current workload in relation to the total memory volume (RAM+swap).

The memory usage graph shows the difference in memory consumption of VisTAS and the standard system. VisTAS generates dynamic codes and uses distributed ledger application, which is highlighted as slightly extra memory usage.

### Results summary

**Figure 11:**
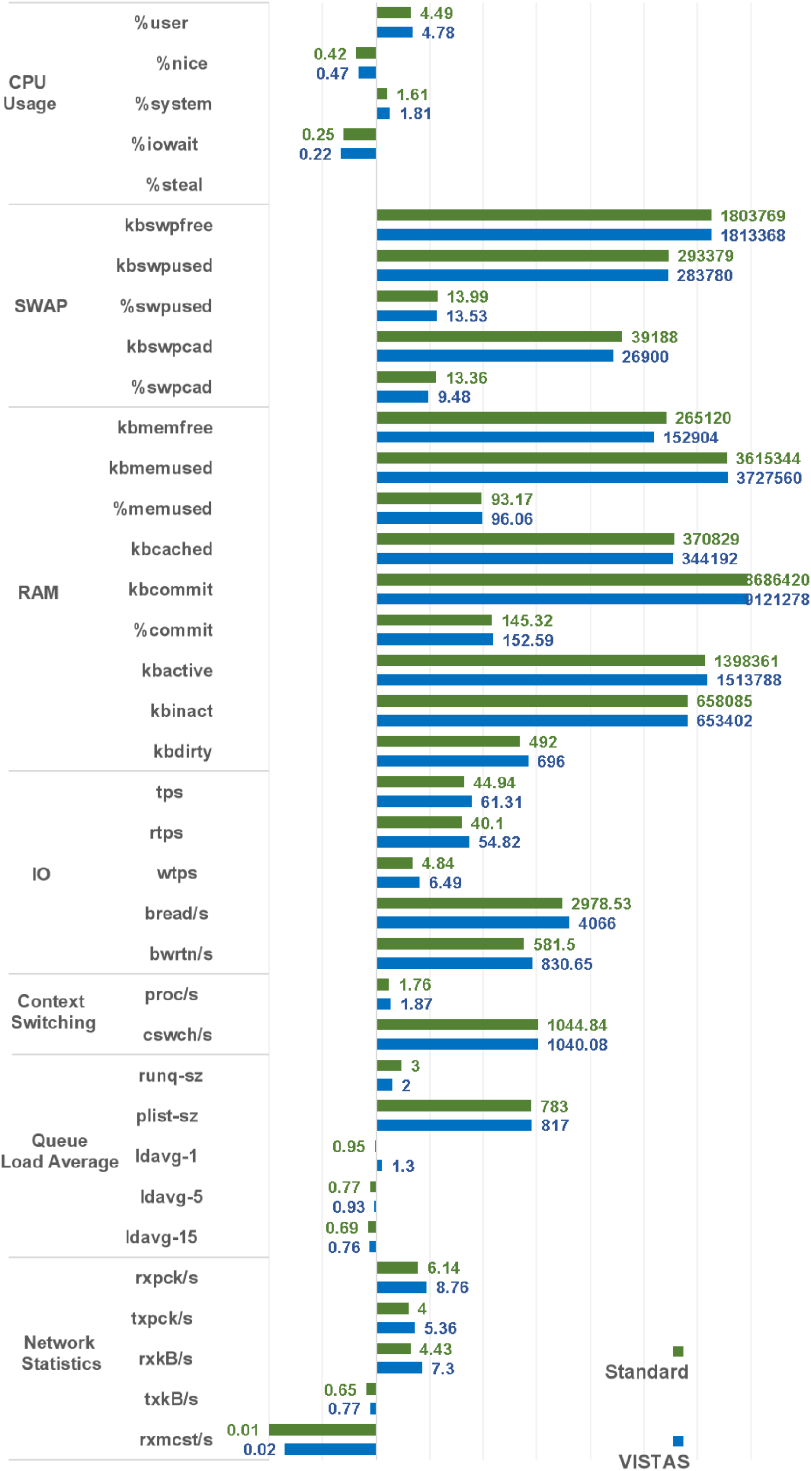
Overall SAR analysis of the VisTAS.

A comparison graph of these performance matrices is depicted in Fig. 11. By evaluating and analyzing these values, we have achieved deterrence, peer control, visibility over the remote authentications, and immutable record of login requests.

## SWOT Analysis

Linux is a well-known OS rendering various servers and cloud services. It has different flavours, including Redhat, Ubuntu, CentOS, Fedora, SUSE and Oracle Linux, etc. CentOS (Community Enterprise Operating System) and Oracle Linux servers are Redhat extensions. For the implementation of the VisTAS, on the server-side, freely available open-source CentOS Server 8.0,86_64 Operating System and for distributed ledgers and immutability, we commissioned Multichain (Multichain, 2019), which is an open-source implementation of the bitcoin protocol. On the client-side, we used command-line applications like Linux terminal and putty. Linux provides Plug-able Authentication Module (PAM) to implement an identification and authentication framework. VisTAS exploited this functionality positively to provide mutual authentication, as shown in Fig. 3.

**Figure 12:**
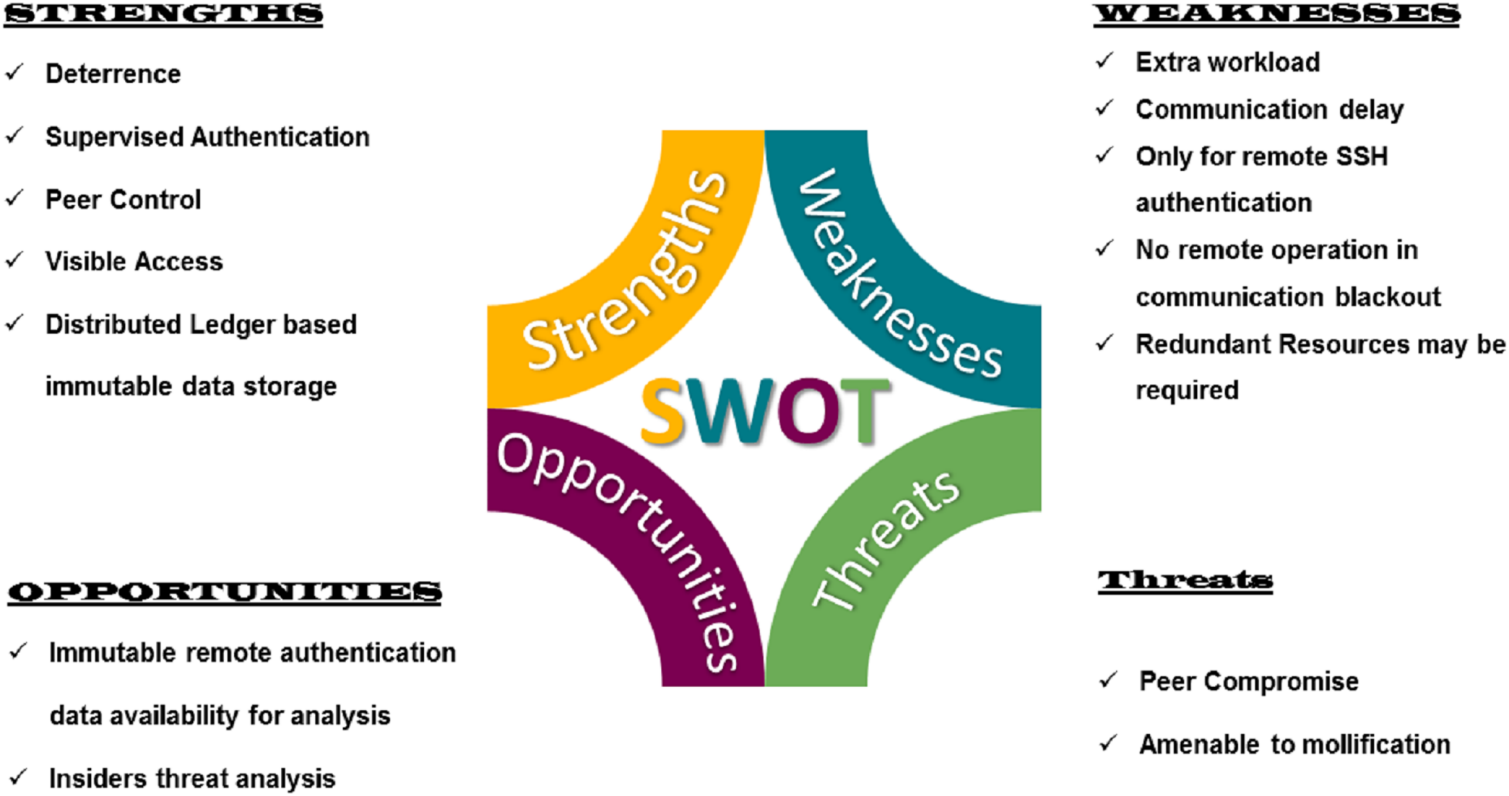
SWOT analysis of the proposed scheme.

The proposed model provided a mechanism for remote authentication, which has it’s good and bad. Based on the graph presenting the overall impact of VisTAS Fig. 11 as discussed earlier in previous sections, SWOT analysis Fig. 12 is used to analyse and highlight the good and the bad. The proposed model provides 4Ds out of the well-known 5Ds of Security, i.e., Deter, Detect, Delay, Deny and Defend.

### Strengths

Deterrence, Delay, Deny and Defend are the strengths of the proposed model. Deterrence is spelt out by providing visibility to the peers and recording authentication data to the blockchain. The delay factor is achieved by getting peer permission for authentication. The peer can deny authentication if she smells some insider threat, and overall, the defence is achieved by rejecting all the illegal authentication requests.

### Weaknesses

The system has to face some extra processing workload of blockchain and dynamic key transfer. Similarly, communication delay may also occur in peak working hours where network congestion may delay the legitimate requests and remote administrative operation or activity can not be performed in case of communication blackout.

### Opportunities

Detection of insider threat is possible only if legitimate data of authentication schemes are available. Using this scheme, we can analyse this data to detect the insider and achieve the 5th “D” of security.

### Threats

Peers and stakeholders are equally responsible for the success of any security system. In this scheme, peers are actively involved and may pose some threat of blackmailing or unavailability of services.

### Results summary and discussion

This framework’s effectiveness and workload are obtained by monitoring the server on multiple parameters, especially the usage of processing power, I/O activities, Random Access Memory (RAM), network utilisation, swap memory, and context switching. It is a win-win situation if the system/facility becomes deterrent/secure without compromising these performance matrices significantly. There are multiple application and system monitoring tools freely available, like System Activity Reports (SAR) in Linux. Comparisons of these performance matrices are discussed in the following paragraphs.

This research used the SAR utility to generate performance comparison on standard authentication viz-a-viz VisTAS based authentication. We focused on monitoring only necessary performance matrices like CPU usage, memory usage, I/O load, swap memory usage, context switching, process queue, and network traffic only.

**I/O Analysis:** The Input-Output (I/O) overhead significantly affects the performance of a system. SAR statistics in terms of I/O and transfer rate shows marginal overhead.
**CPU Usage Analysis:** By analysing the graph’s values, the processing impact is very minute while using VisTAS compared to standard authentication.
**Context Switching Analysis:** The Context Switching leads to an extra workload due to sharing the system cache among multiple tasks and running the scheduler etc. Context switching between threads of the same application or process is faster than among different processes. Its overhead can be monitored by processes created in a per unit of time, and the number of context switching occurred per second as following.
**Process Queue Analysis:** Statistics showing the length of the system process’s queue and load average determine the system’s efficiency. The following can be used to check the system’s health and performance: The impact of VisTAS on process queue management compared to standard authentication as depicted in queue-management is negligible.
**Network Traffic Analysis:** While comparing network load between VisTAS and standard authentication, as shown in network load, it observed a difference of two to three packets for an authentication activity.
**Swap Memory Usage Analysis:** Swap memory extends system memory. The utilisation of this memory is also used to monitor the system’s performance. Swap memory load shows the swap memory impact comparison between VisTAS and standard authentication and ignore-able difference found swap memory utilisation. The memory usage graph shows the difference in memory consumption of VisTAS vs standard system. VisTAS generates dynamic codes, uses a distributed ledger application, and consumes slightly extra memory, which is highlighted in the swap memory usage analysis graph in Fig. 9.

## Conclusion & Future Work

Insider threats are of primary concern for all types of organizations. Extensive research has been carried out in this domain of information security. The deterrence perspective is missing for insiders in information security because of their specialised privileges to the system resources. Insider threat mitigation strategies in the dimensions of the social, behavioural, and technical arena are explored.

Shared Secret, any two of three and two men rule, etc., have already been used and practised for a long time in physical security measures such as physical locks for significant and critical sites. Implementing the two-person rule would strengthen the information system’s protection and visibility in its operations by authenticating and validating respective stakeholders’ activities. This paper suggested a two-person approach with a supervisory concept for authentication and validation of user activities. A pre-arranged scenario is also discussed with a view to practice this system. The system should therefore deal with increased processing due to the blockchain utilisation and dynamic key transfer. Similarly, during peak working hours, network congestion may cause legitimate requests to get delayed. In the case of a communication blackout, remote administrative operations or activities cannot be performed.

Implementation of VisTAS revealed that specifically for mission-critical environments like data centres/Supervisory Control and Data Acquisition Systems (SCADA), the framework is quite effective and renders a practical approach. Performance evaluation of VisTAS shows satisfactory and competitive results. In the future, a rigorous assessment is planned in which stress tests will be carried out by deploying the framework into some large-scale environment for validation purposes.

We have implemented VisTAS in a test-bed of the Linux environment, and its performance has been evaluated using SAR. According to the results depicted by SAR, additional processing is required for extra security. In our future work, we will implement this framework in a suitable open-source Operating System to facilitate the implementation of the proposed framework and find interlinking user activities through some graph databases.

## Supplemental Information

10.7717/peerj-cs.516/supp-1 Supplemental Information 1 VisTAS Implementation. Script and configuration source code for the implementation of VisTAS

## Additional Information and Declarations

### Data Availability

The following information was supplied regarding data availability: Code and instructions are available in a Supplemental File.
